# Superlattice growth and rearrangement during evaporation-induced nanoparticle self-assembly

**DOI:** 10.1038/s41598-017-02121-4

**Published:** 2017-06-05

**Authors:** Elisabeth Josten, Erik Wetterskog, Artur Glavic, Peter Boesecke, Artem Feoktystov, Elke Brauweiler-Reuters, Ulrich Rücker, German Salazar-Alvarez, Thomas Brückel, Lennart Bergström

**Affiliations:** 1 Jülich Centre for Neutron Science (JCNS) and Peter Grünberg Institute (PGI), JARA-FIT, Forschungszentrum Jülich GmbH, 52425 Jülich, Germany; 2 Institute of Ion Beam Physics and Materials Research, Helmholtz-Zentrum Dresden Rossendorf, 01328 Dresden, Germany; 3 Department of Materials and Environmental Chemistry, Stockholm University, 10691 Stockholm, Sweden; 4 Department of Engineering Sciences, Angström Laboratory, Uppsala University, 751 21 Uppsala, Sweden; 5 Laboratory for Neutron Scattering and Imaging, Paul Scherrer Institut, 5232 Villigen PSI, Switzerland; 6 ESRF-The European Synchrotron, 38043 Grenoble, France; 7 Jülich Centre for Neutron Science (JCNS) at Heinz Maier-Leibnitz Zentrum (MLZ), Forschungszentrum Jülich, 85747 Garching, Germany; 8 Institute for Complex Systems, Bioelectronics (ICS-8), Forschungszentrum Jülich GmbH, 52425 Jülich, Germany

## Abstract

Understanding the assembly of nanoparticles into superlattices with well-defined morphology and structure is technologically important but challenging as it requires novel combinations of *in-situ* methods with suitable spatial and temporal resolution. In this study, we have followed evaporation-induced assembly during drop casting of superparamagnetic, oleate-capped *γ*-Fe_2_O_3_ nanospheres dispersed in toluene in real time with Grazing Incidence Small Angle X-ray Scattering (GISAXS) in combination with droplet height measurements and direct observation of the dispersion. The scattering data was evaluated with a novel method that yielded time-dependent information of the relative ratio of ordered (coherent) and disordered particles (incoherent scattering intensities), superlattice tilt angles, lattice constants, and lattice constant distributions. We find that the onset of superlattice growth in the drying drop is associated with the movement of a drying front across the surface of the droplet. We couple the rapid formation of large, highly ordered superlattices to the capillary-induced fluid flow. Further evaporation of interstitital solvent results in a slow contraction of the superlattice. The distribution of lattice parameters and tilt angles was significantly larger for superlattices prepared by fast evaporation compared to slow evaporation of the solvent.

## Introduction

Nanoparticle superlattices consisting of dense packed particle assemblies with periodic arrangements and tunable spacings are of interest for magnetic, plasmonic and optolectronic applications^1–6^. Robust methods to produce nanoparticles with controlled compositions, sizes, and shapes^7–11^ have enabled the production of nanoparticle superlattices with a wide range of structures and compositions^2, 12–15^. There are many methods to produce nanoparticle superlattices, but evaporation induced self-assembly on solid substrates (drop casting) is arguably the most widely used^16–20^. Self-assembly of nanoparticles from a dilute dispersion by drop casting is a complex process that is controlled by the particle interactions, packing constraints, mass and heat transfer during drying, together with kinetic and thermodynamic factors^21–24^.

While the structural diversity of the nanoparticle assemblies is beginning to unravel, the understanding of how the nanoparticle arrays form is poorly developed. Measuring the dynamics of assembling nanoparticles in liquids is challenging and requires not only methods with suitable spatial- and time-resolution, but also the design of measurement environments where e.g. the evaporation rate can be controlled^25^. Light microscopy^16, 26^ and liquid-cell electron microscopy^27–29^ have provided important insight into evaporation-driven phenomena on large and small lengths scales, respectively. Small Angle X-ray Scattering (SAXS) and Grazing Incidence SAXS (GISAXS) have recently evolved to be the methods of choice to obtain time-dependent information on the assembly process^17, 18, 20, 22, 30–37^. The main strength of scattering experiments compared to local probes is the unique combination of resolution and statistics as they provide the relevant ensemble averages directly.

Recent work using GISAXS has shown that nanoparticle superlattices may undergo time-dependent structural transitions during solvent evaporation^37, 38^ and that the separation distance in DNA-capped nanoparticle assemblies can be reversibly increased or decreased by dehydration and rehydration^39^. Routh recently reviewed how the structural transitions and cracking during drying of colloidal films are related to fluid flow and the dynamics of capillary pressure gradients^40^. Previous work has indeed shown that the evaporation-induced assembly process can be strongly influenced by the fluid flow and convective particle transport, including e.g. coffee-ring effects^41^ and Marangoni flows^42^. However, there is lack of information on how the evaporation rate and drying front movement control the structural evolution of nanoparticle superlattices during drop casting.

In this study, we follow the evaporation induced self-assembly of iron oxide nanoparticle superlattices by real-time *in-situ* small angle X-ray scattering and simultaneously the droplet height and drying front movement, in a cell with controlled environment. An automatized fitting routine enabled us to obtain quantitative information from thousands of GISAXS patterns, and provided time-dependent data on the unit cell size, the orientation of the unit cell, and the distribution of lattice constants of the self-assembled superlattices. The stability and simplicity of this novel fitting method, utilizing multiple coupled peaks for redundancy, yields readily information from large sets of *in-situ* GISAXS measurements. The combination of X-ray scattering, light band micrometer measurements and imaging of the drop surface allows us to identify different growth stages during drop casting and to couple the onset of superlattice formation to the movement of a drying front across the drying droplet. Quantification of the coherent and incoherent scattering intensity, describing the number ratio of ordered and disordered particles, provides information on the kinetics of superlattice growth. The novel method for evaluating large sets of GISAXS data and the importance of the drying front for superlattice growth can be used for better control of the self-assembly process to enable rapid production of nanoparticle superlattices.

## Results and Discussion

**Figure 1:**
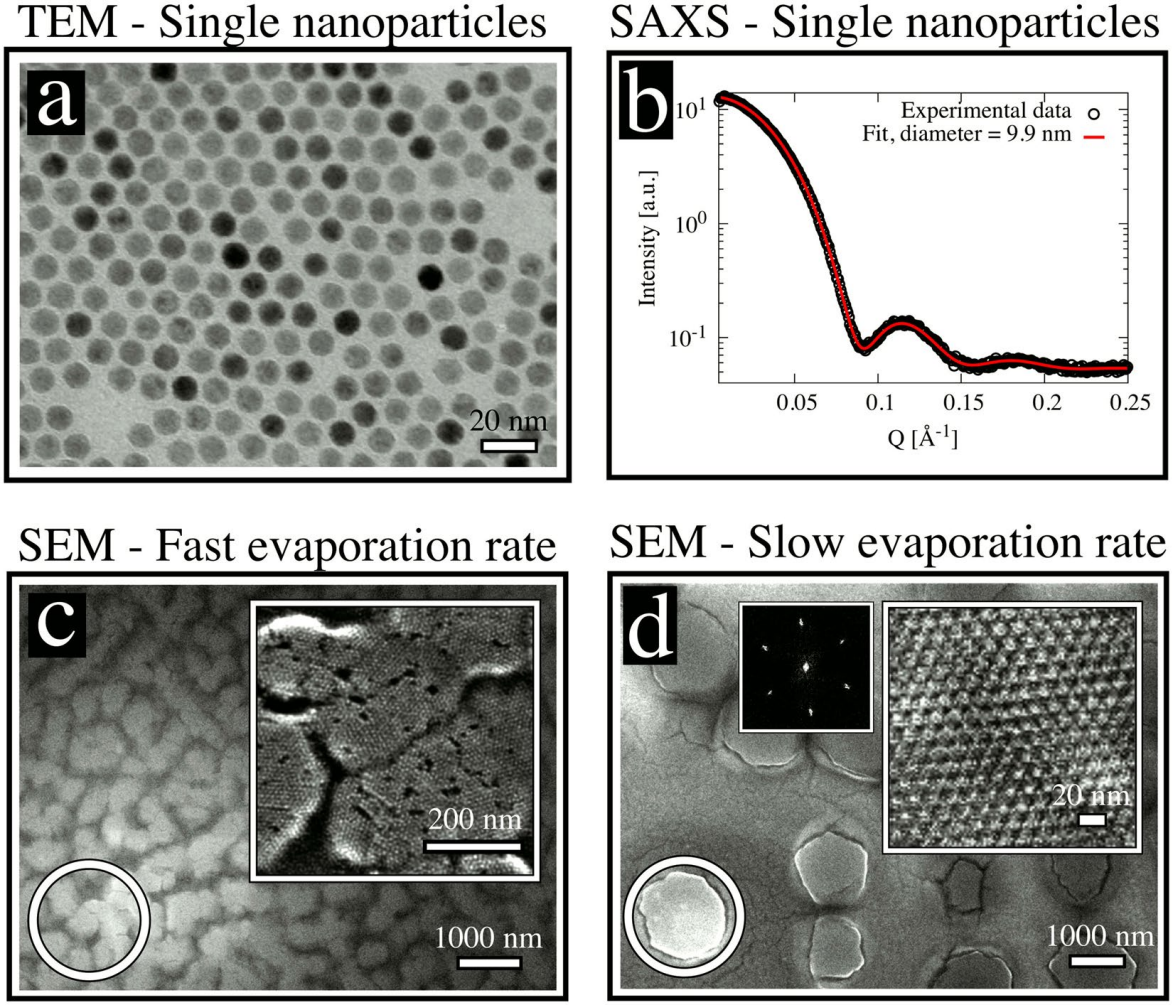
Characterisation of nanoparticles and superlattices. (**a**) TEM image of monolayer of spherical iron oxide nanocrystals. (**b**) SAXS pattern of a dilute nanoparticle dispersion together with the fit to the data with a spherical form factor. (**c**,**d**) SEM images of 3D nanoparticle superlattices produced at (**c**) *fast* and, (**d**) *slow* evaporation rates. The inset on the right corner shows a higher magnification of the marked area (circle). The inset on the left side of (**d**) is a fast Fourier transformation (FFT) of the superlattice.

**Figure 2:**
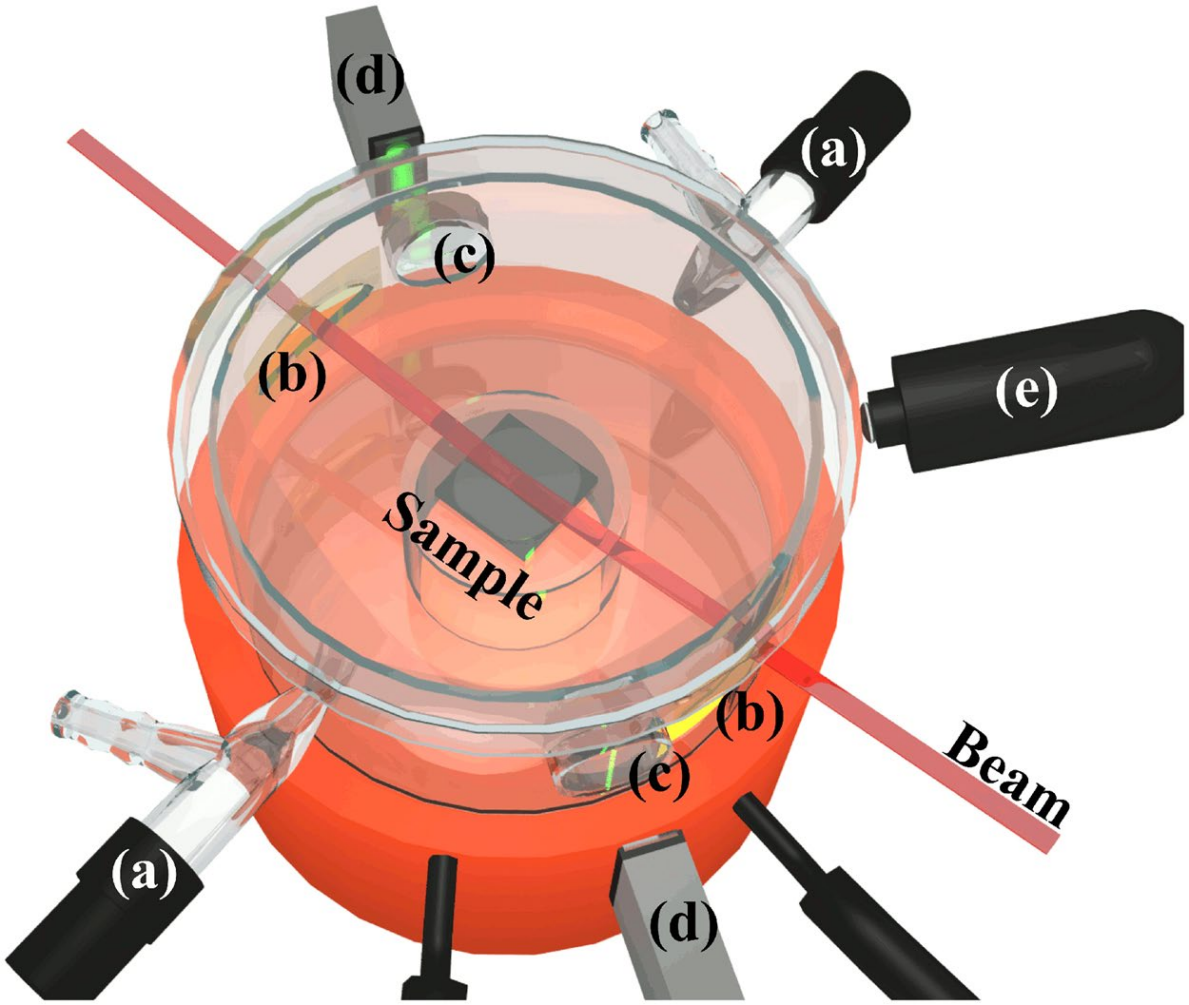
Illustration of the evaporation cell. Schematic outline of the evaporation cell used for the in-situ SAXS and GISAXS measurements. The cell is equipped with gas flow control (**a**), Kapton windows (**b**), glass windows (**c**), light band micrometer (**d**), and camera (**e**).

**Figure 3:**
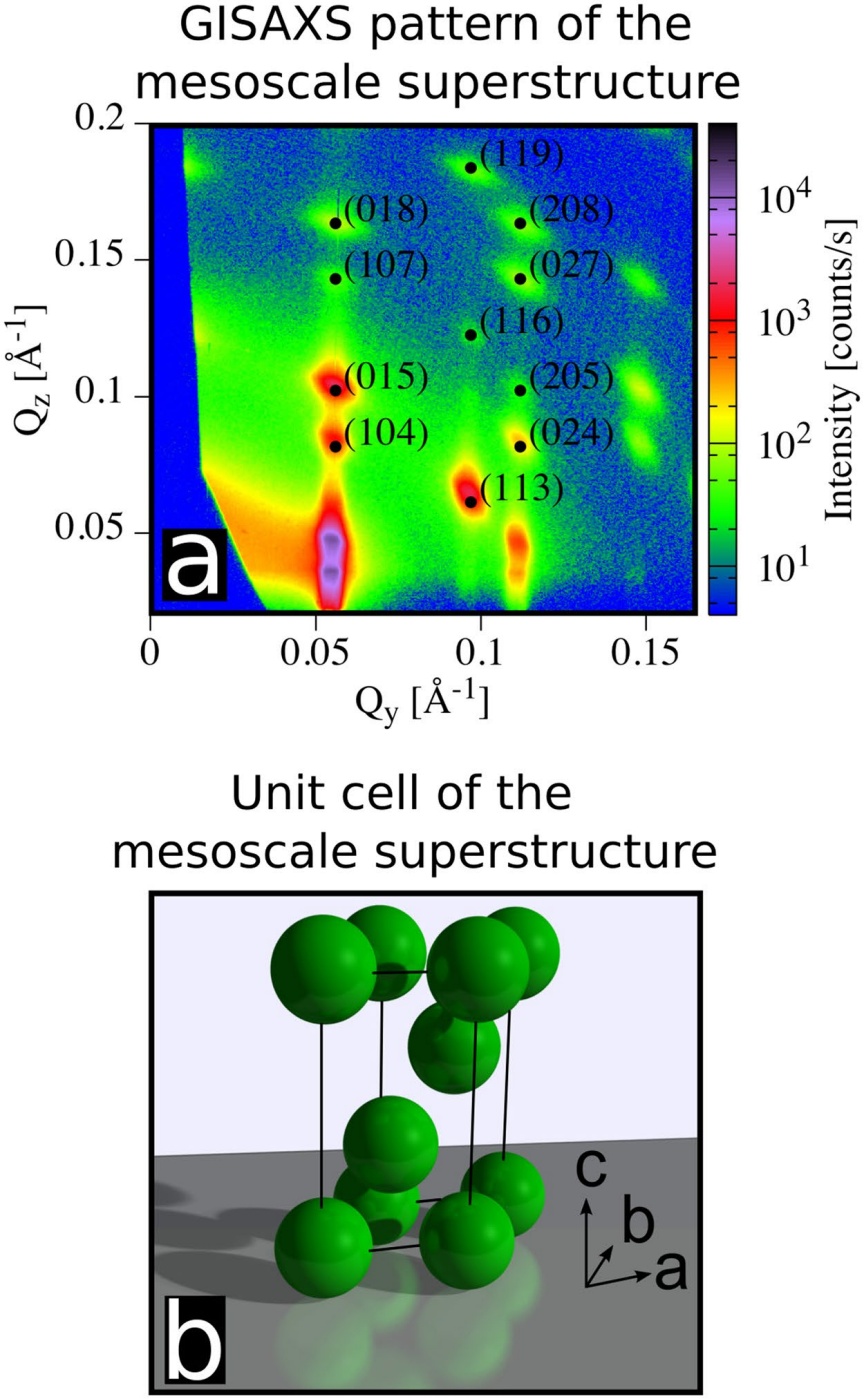
GISAXS pattern and unit cell structure of the drop cast nanoparticle assemblies formed by *slow* evaporation. (**a**) Selected well-defined peaks of the GISAXS pattern are indexed to a R$\bar{3}$m structure, which represents the final structure for all investigated samples. **(b)** Illustration of the rhombrohedral unit cell as defined by the GISAXS analysis (see Fig. 6 for unit cell parameters).

Spherical iron oxide nanoparticles (Fig. 1a) were synthesized by thermal decomposition of iron-oleate^43^. Analysis of SAXS data on dilute dispersions yields an average inorganic core diameter of 9.9 ± 0.1 nm and a size distribution of 6.4 ± 0.5% (Fig. 1b)^44^. Drop cast assemblies of the oleate-capped spherical nanoparticles dispersed in toluene were formed by controlled evaporation of the drops deposited onto a Si wafer in a custom-built evaporation cell (Fig. 2). Assemblies formed at *slow* evaporation rate (relating to an average decrease rate of the droplet height of 0.07 *μ*m/s) displayed a high degree of long-range order (Fig. 1d). The *quickly* evaporated nanoparticle dispersion (relating to an average decrease rate of the droplet height of 0.7 *μ*m/s) forms highly defective assemblies that lack a long-range order beyond the domain size of about ≈100 nm (Fig. 1c). Structural analysis by GISAXS shows that all particle assemblies display a rhombohedral structure with the space group R$\bar{3}$m (see Fig. 3a for indexing and Fig. 3b for the unit cell), irrespective of the evaporation rate. The full indexing of the peaks is shown on a sample produced under similar conditions in a previous publication^15^. The analysis of the space group is done according to well established crystallographic methods, but with the special case that all reflections hkl from different directions arise in one single GISAXS pattern without any rotation of the sample^19^, as in a powder sample, but with additional directional information for the out-of-plane axis. In other words, the 2D powder leads to reciprocal space rings in the Qx-Qy plane and the experiment measures one slice through this plane. The pure (001) reflections have a ring radius of 0 and therefore lead to much stronger reflections in the measured plane. The flat Ewald sphere in small angle scattering, as well as the 2D powder property with the in-plane orientation average induce this phenomenon. It should be noted that the magnetic field will induce a weak attraction between the nanoparticles that promote the formation of ordered assemblies but is too weak to induce the formation of strings or other tenuous structures^12^.

We have followed the structural evolution during evoporation-induced self-assembly by SAXS and GISAXS. Previous studies^16, 17, 45^ have shown that assembly can occur at both, the solid-liquid and the air-liquid interface. To investigate these possibilities, we performed transmission SAXS measurements that probed the air-liquid interface as the droplet height decreased from an initial height of about 300 *μ*m down to a height of 50 *μ*m. Independent of the evaporation rate, we did not observe any sign of ordered clusters in the bulk or at the air-liquid interface (Fig. SI-3). Estimates of the diffusion rate of the oleate-capped nanospheres (with a hydrodynamic diameter d_*H*_ ≈ 14 nm) in a dilute toluene dispersion, and the velocity with which the air-liquid interfaces move during evaporation show that they are of similar magnitude for the *fast* evaporation, and that diffusion dominates at the *slow* evaporation rate. This suggests that particle capture at the liquid-air interface is unlikely at the slow evaporation rate and probably very rare at the fast evaporation rate. A weak magnetic field has been applied with a gradient along the surface normal upwards because previous studies showed an optimal order. The field is not strong enough to promote the superparamagnetic nanoparticles to be transported to the solid-liquid interface.

**Figure 4:**
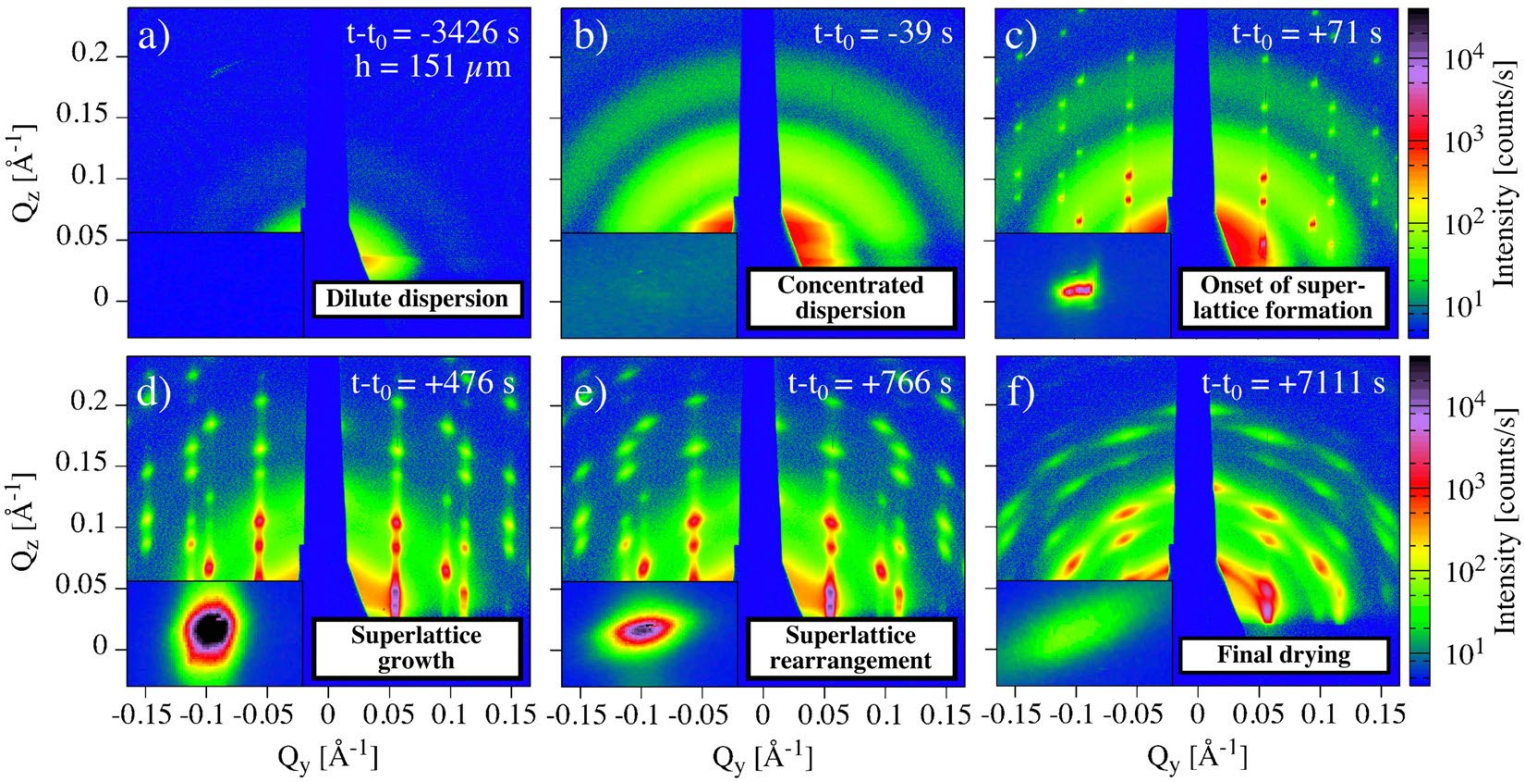
Time-resolved X-ray scattering reveals the transition from a disordered nanoparticle dispersion to a highly ordered superlattice. Temporal evolution of GISAXS patterns during evaporation-induced self-assembly of iron oxide nanospheres at the *slow* evaporation rate. The label at the upper right corner indicates the drying time in relation to the time t_0_, when first structure peaks appear, and the droplet height, h, (only for (**a**)). The patterns (**a**–**f**) illustrate the transition from non-interacting nanoparticles in a dilute dispersion to highly ordered particle assemblies after evaporation of the carried solvent. The insets in each image show a detailed view of the (0–15) reflex on a linear color scale (see Fig. 3a for indexing).

Results from the time-dependent *in-situ* GISAXS study during drop casting at the *slow* evaporation rate are shown in Fig. 4. The initial stage (Fig. 4a) is characterized by a feature-less scattering pattern, relating primarily to the form factor of the non-interacting nanospheres. As evaporation progresses and the particle concentration increases (Fig. 4b), the intensity of the form factor rings gradually increases. This process continues until sharp peaks suddenly appear (Fig. 4c), which signifies the onset of superlattice formation. The sharp peaks develop in full within two sequential measurements (spaced by 7 s) i.e. much faster than the time resolution of the instrument.

The Bragg peaks are very sharp, suggesting that the ordered superlattices have a large size from the onset of growth. Continued growth of the amount of superlattices results in an increase of the peak intensity and peak width over a period of ≈10 min at the *slow* evaporation rate (cf. Fig. 4c and d). Conversely, the continuous growth of the superlattices causes a reduction of the intensity of the incoherent background over time.

As evaporation progresses, we observe that the peak shape becomes increasingly diffuse (cf. Fig. 4d and e). This suggests that the distribution of lattice constants between the mesoscopic superstructures broadens and the crystals develop a random tilt around the surface normal. Prior to terminating the experiment, the cell was opened to allow the remaining toluene to evaporate. The final drying of the solvent saturated arrays resulted in significant broadening of the peaks, suggesting that the final drying induces significant distortion and inhomogeneous shrinkage of the superlattices (Fig. 4f).

**Figure 5:**
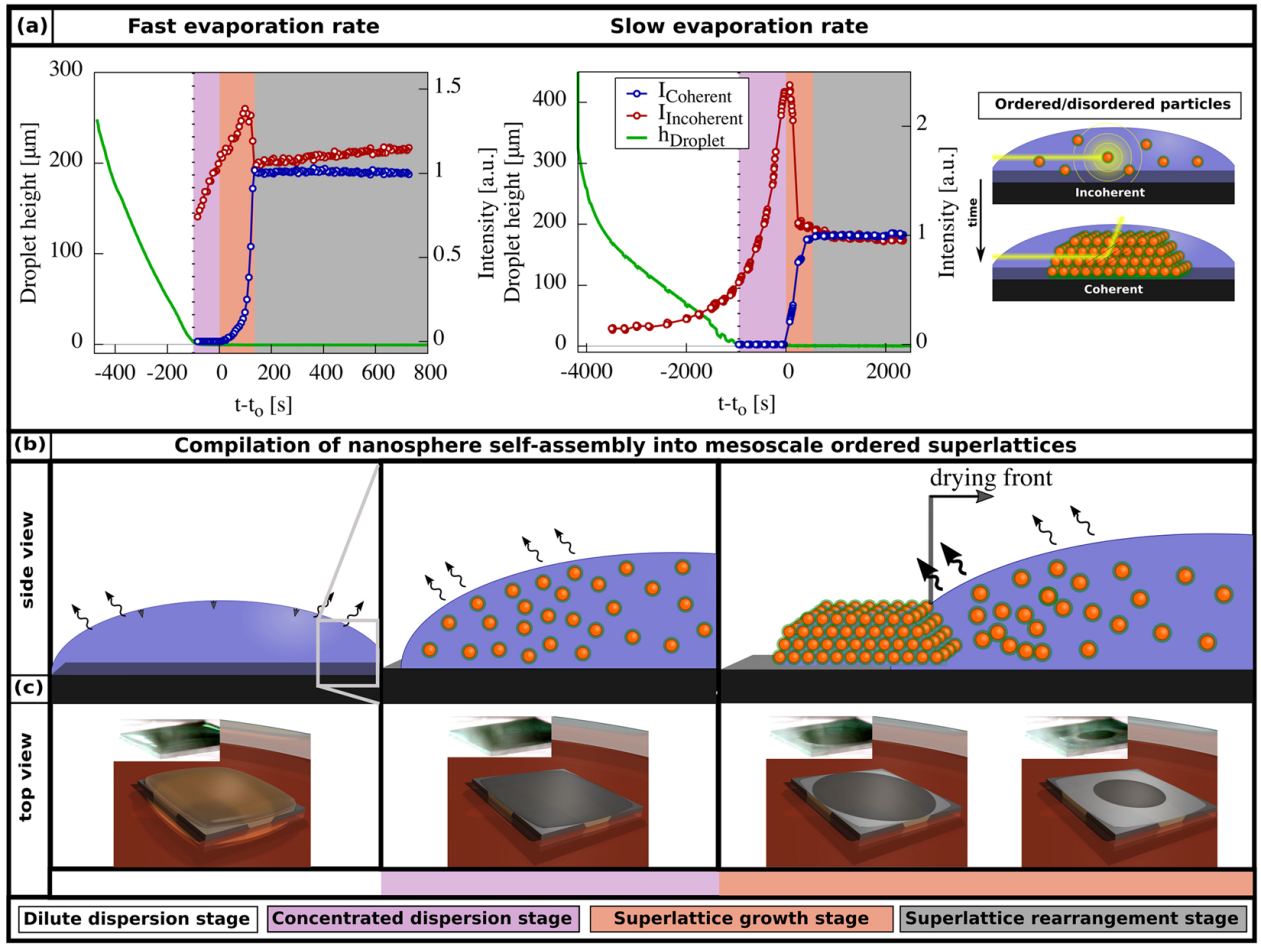
Time-dependent GISAXS scattering intensity information and drying behaviour identify the temporal transitions during drop casting. (**a**) Time-dependent evolution of the coherent and incoherent scattering intensity together with the height of the droplet for the *slow* and *fast* evaporation rate experiments. The major stages during drop casting are indicated by the colored regions in the background. The value t_0_ describes the onset of superlattice formation, i.e. the time when a detectable coherent intensity first evolves. (**b**) Schematic illustration of how the transition from a concentrated dispersion to the onset of superlattice formation is related to, (**c**) the occurrence and movement of a drying front across the surface of the thin nanoparticle dispersion film. The insets show photos of the top surface of the drying droplet obtained at different times during drop casting.

The GISAXS data was processed using a software developed in-house (see SI for further details)^46^. The software enables rapid analysis of a large number of GISAXS data sets. This yields quantitative information on the time-resolved evolution of lattice constants, lattice constant distributions and tilt angles of the nanoparticle superlattices with respect to the substrate. Moreover, by quantification of the coherent and incoherent intensities, it is possible to quantify the relative ratio of nanoparticles in ordered assemblies/superlattices (see Fig. 5a for schematic illustration). Figure 5 displays a detailed analysis of the four major stages during drop casting of nanoparticle superlattices: *dilute dispersion*, *concentrated dispersion*, *superlattice formation and growth*, *and superlattice rearrangement stage*.

The first stage (*dilute dispersion*) is marked by the white area in Fig. 5. The droplet height decreases continuously and linearly at both *slow* and *fast* evaporation rates. The dilute dispersion stage is characterised by noninteracting nanoparticles (Fig. 5b), resulting in scattering intensities determined purely by the individual nanoparticle form factor (Fig. 1).

The second stage, the *concentrated dispersion stage* (light purple area) is characterized by a non-linear increase of incoherent scattering intensity (cf. red data points in Fig. 5a) as evaporation progresses and a large number of nanoparticles move into the synchrotron radiation beam. The film thickness is so small that it cannot be measured accurately by the light band micrometer^26^ but the camera images show that the liquid film is continuous (Fig. 5c).

We found that the duration of the concentrated dispersion stage varies with the initial evaporation rate of the droplet. For the *fast* experiment this stage exists for ≈1.5 min in contrast to ≈15 min for the *slow* experiment. The strong reduction of the evaporation rate during the growth stage is probably related to the gradual enrichment in the thin film^47^.

The onset of the *superlattice formation stage* (light red area) is characterized by the rapid increase of the intensity of the coherent scattering (Fig. 5a) and the emergence of a drying front that originates at the three-phase contact line and recedes inwards towards the substrate centre (inset in Figs 5c and SI-4 shows a more detailed view on the movement of the drying front). Simultaneously, for the *fast* evaporation rate the intensity of the incoherent scattering first increases and then decreases within this stage (Fig. 5a red points). In contrast, for the *slow* evaporation rate we monitor only a decrease of the incoherent scattering intensity. The occurrence of a drying front marks the interface between saturated solution and partially dry regions and is also related to the build up of a capillary pressure in the partially saturated region^40^.

Although it is not possible to obtain an accurate measure of the height of the droplet, as the height is smaller than the smallest measurable film thickness (5 *μ*m), we can estimate the critical nanoparticle concentration at the time of nucleation to ≈10^17^ NPs per ml (1000 mg/ml) by assuming that the film thickness is on the order of the height of the superlattices (around 500 nm).

The time window for superlattice growth, i.e. the time between t_0_ and when I_*coherent*_ has reached its maximum value, is ≈3 min for the *fast* and ≈9 min for the *slow* evaporation rates, respectively. We find that these times correspond well to the time the drying front needs to move from the edge to the centre of the beam footprint. The drying front is associated with the build up of a capillary pressure. The capillary pressure will assist in pushing the oleate-capped iron oxide nanospheres closer together and promote the rapid formation of large, highly ordered 3D superlattices. The ability of drying fronts to consolidate soft particles into solid films is well known from studies on polymeric coatings^40^. The capillary pressure gradients during drying can also result in cracking of the films, which can be observed in Fig. 1d.

Detailed analysis of the time-dependent GISAXS data yields information on how the structural parameters evolve during the *superlattice growth stage* and the *superlattice rearrangement stage*.

**Figure 6:**
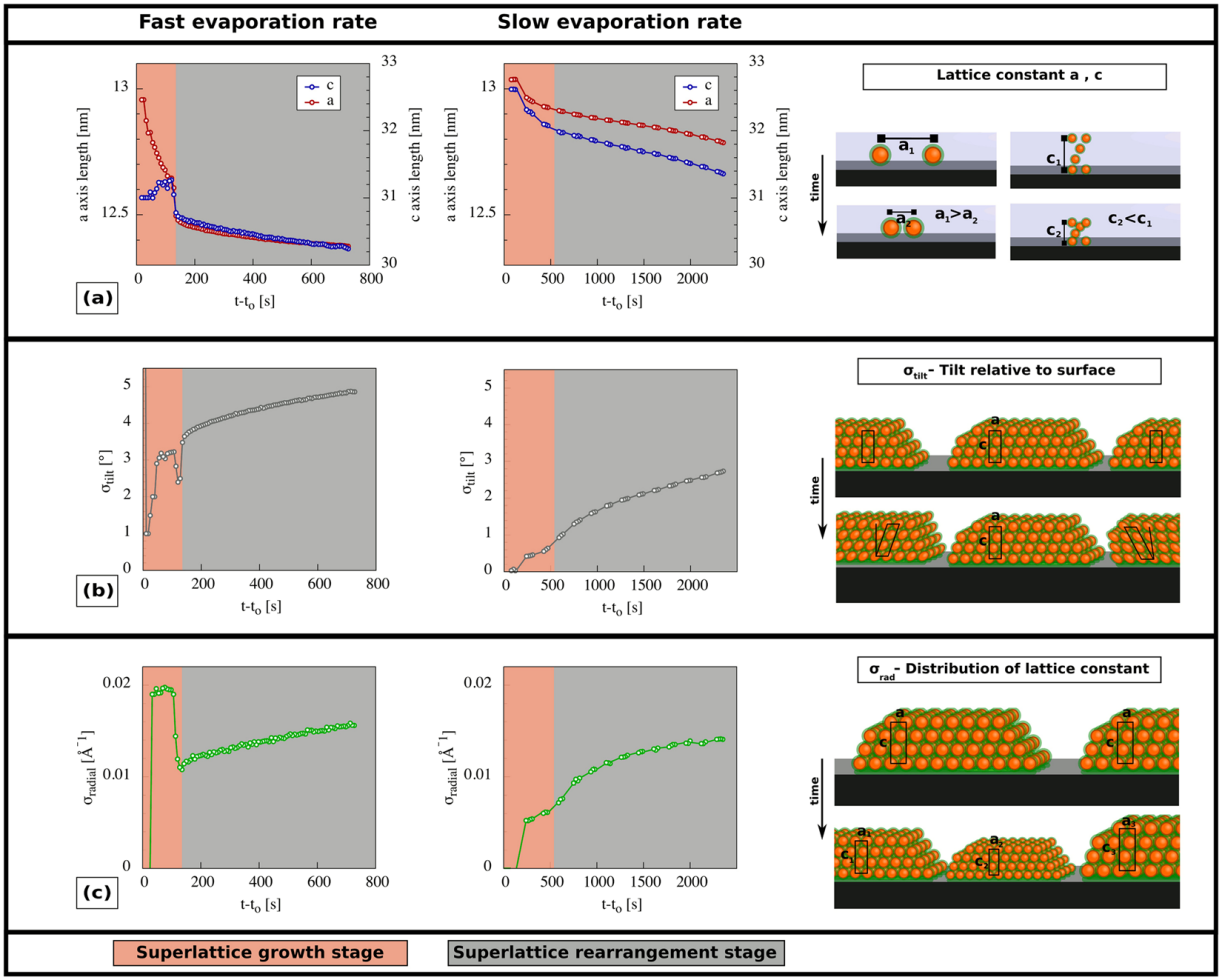
Structural transitions during superlattice growth and rearrangement. Time-dependent changes during drop casting at *fast* and *slow* evaporation rates of; **(a)** lattice constants; **(b)** unit cell tilt; and **(c)** distribution of lattice cell constants of the superlattices.

Drop casting at the *slow* evaporation rate results in a superlattice that continuously shrinks as the solvent evaporates (Fig. 6a). Drop casting at the *fast* evaporation rate, however, results in a superlattice where the *a* axis length shrinks but the c axis length first expands and then shrinks rapidly during the superlattice growth stage. The discontinuity of the temporal evolution of the *c*-axis is reflected in similar discontinuities of the tilt angle of the 3D nanoparticle superlattices with respect to the substrate (*σ*_*tilt*_, Fig. 6b) and distribution of the lattice constants (*σ*_*rad*_, see Fig. 6c) during the superlattice growth stage. We speculate that the rapid movement of the drying front and the associated capillary pressure gradient that develops at the *fast* evaporation rate results in the formation of superlattices with a structure that undergoes non-isotropic structural relaxations during the first minutes after formation. Indeed, the rapid narrowing of *σ*_*rad*_ at the end of the growth stage suggests that the equilibration phase is completed as the drying front has passed over the entire substrate.

The lattice constant distribution is much narrower for the superlattices dried at the *slow* compared to the *fast* evaporation rate. This is probably a result of the more homogeneous distribution of superlattice states within the beam footprint at the *slow* compared to the *fast* solvent evaporation rate.

During the *superlattice rearrangement stage* (represented by the grey area) no further growth is observed (*I*_*Coherent*_/I_*Incoherent*_) ≈ constant) (Fig. 5a). During this stage interstitial toluene between the particles evaporates and the distance between the particles decreases monotonously (cf. Fig. 6a grey area). Likewise, the distribution of lattice constants (*σ*_*rad*_) (cf. Fig. 6c) and superlattice tilt angles (cf. Fig. 6b) increase monotonously in both the *slow* and *fast* experiment.

The final particle distances (a- and c-axis lengths) are larger for the superlattices assembled at *slow* rates compared to *fast* evaporation rates. At the end of the experiment, the c-axis is slightly contracted with respect to the fcc limit for superlattices assembled at both *slow* and *fast* evaporation rates (see SI Fig. SI-6 for more information).

## Conclusion

In summary, the formation of highly ordered superlattices composed of oleic acid-coated iron oxide nanospheres could be analysed by *in-situ* GISAXS. A software developed in-house allows for the fitting of the peaks of large GISAXS data sets yielding superlattice tilt angles, lattice constants, and lattice constant distributions. It is possible to identify four different stages: *dilute dispersion*, *concentrated dispersion*, *superlattice formation and growth*, *and superlattice rearrangement stage*. We observed the decrease of all lattice constants over time, converging to a near-perfect fcc packing. We found that there is a strong correlation between the formation and growth of nanoparticle superlattices with the movement of the drying front across the dispersion film. The build up of the capillary pressure at the interface between the saturated and partially saturated regions promotes rapid formation of highly ordered superlattices. Tuning the balance between the capillary pressure and the intrinsic particle interactions can be used to produce superlattices with gradient structures and well-defined particle separations. Such a control of the growth of nanoparticle assemblies is demonstrated in this work by varying the evaporation rate.

## Methods

### Synthesis and characterization

Iron oxide nanospheres were synthesized by thermal decomposition of iron-oleate and is described in detail elsewhere^43, 44^. The synthesis results in oleate-capped iron oxide nanospheres dispersed in toluene. The initial particle concentration of the used nanoparticle solution is 8.4 * 10^14^ NPs per ml (2.2 mg/ml). The particle size distribution of the iron oxide nanospheres was determined using a Bruker AXS Nanostar SAXS instrument (Cu K*α*, *λ* = 1.54 *Å*). SEM images were acquired with Magellan 400 SEM (FEI). The high resolution images were FFT-filtered for clarity.

### Evaporation-induced assembly (drop casting)

The drop casting experiments were performed by allowing a 20 *μ*l droplet of a dilute nanoparticle dispersion, deposited on a silicon wafer (1 cm^2^) to slowly evaporate inside a specifically designed evaporation cell (Fig. 2). The silicon wafer was cleaned by subsequent sonication in ethylacetate and ethanol, and then dried prior to use. All drop casting experiments were performed under a weak magnetic field that was applied perpendicular to the substrate (B = 360 G) with a vertical gradient dB/dz = 80 G/cm. The evaporation rate in the cell was controlled by the flow of nitrogen through the inlet/outlet valves (Fig. 2a) and a toluene reservoir that was placed close to the silicon substrate. The evaporation/measurement cell was equipped with Kapton windows (Fig. 2b) to allow the transmission of X-rays with a large scattering angle of up to 16°. The height of the droplet was followed with a nominal resolution of 0.1 *μ*m using a light band micrometer (Keyence, Fig. 2d), consisting of a strong diode that emits a monochromatic, line-shaped beam, and a high-resolution camera that capture the light band image. The light was transmitted through two flat windows (Fig. 2c), offset by 30° against the X-ray beam axis. The droplet was photographed using a macrophotography camera (Fig. 2e). The gas flow was adjusted to perform the drop casting experiments at two evaporation rates, which related an average decrease of the droplet height (dh/dt) with 0.7 *μ*m/s for the *fast* evaporation rate experiments (called *fast*) and dh/dt = 0.07 *μ*m/s for the *slow* evaporation rate experiments (called *slow*).

### SAXS and GISAXS

The *in-situ* SAXS and GISAXS measurements were carried out at the ID01 beamline at the European Synchrotron Radiation Facility (ESRF). All SAXS and GISAXS experiments were performed at 9.8 keV photon energy. During the initial evaporation stage, transmission SAXS measurements (i.e. measurements through the droplet) were performed at several different heights for the *slow* evaporation rate experiments, and at a single position close to the substrate for the *fast* evaporation experiments (see SI for details). The GISAXS measurements were performed at an angle of *θ* = 0.3°, with a beam size corresponding to a footprint of 1mm horizontal by 50 *μ*m vertical. The different scattering geometries are shown in Fig. SI-1. GISAXS data was acquired with intervals of 7 seconds at a 1 second exposure time, limited by the read out of the area gas detector. The quantitative analysis of the time-dependent scattering patterns was performed using a software developed in-house (see description in the SI)^46^. In short, the software is able to fit automatically a large sequence of GISAXS data sets. The model of fit functions used is in very good agreement with the data measured (see Fig. SI-2). The software can be accessed for download in the supplement and run under the plot.py software.

### Available software

The software FITin-situGISAXS can be accessed for download at https://github.com/science01/FITin-situGISAXS and run under the plot.py software https://sourceforge.net/projects/plotpy/.

## Electronic supplementary material

Supplementary Information

## References

[1] Claridge SA (2009). Cluster-Assembled Materials. ACS Nano.

[2] Shevchenko EV, Talapin DV, Kotov NA, O’Brien S, Murray CB (2006). Structural diversity in binary nanoparticle superlattices. Nature.

[3] Dreyer A (2016). Organically linked iron oxide nanoparticle supercrystals with exceptional isotropic mechanical properties. Nat. Mater..

[4] Moser A (2002). Magnetic recording: advancing into the future. Phys. D: Appl. Phys.

[5] Reiss G, Hutten H (2005). Magnetic nanoparticles: Applications beyond data storage. Nat. Mater..

[6] Whitham K (2016). Charge transport and localization in atomically coherent quantum dot solids. Nat. Mater..

[7] Glotzer SC, Solomon DJ (2007). Self-assembly: From nanoscale to microscale colloids. Nat. Mater..

[8] Park J (2004). Ultra-large-scale syntheses of monodisperse nanocrystals. Nat. Mater..

[9] Miszta K (2011). Hierarchical self-assembly of suspended branched colloidal nanocrystals into superlattice structures. Nat. Mater..

[10] Dong A, Chen J, Vora PM, Kikkawa JM, Murray CB (2010). Binary nanocrystal superlattice membranes self-assembled at the liquid-air interface. Nature.

[11] Kovalenko MV (2015). Prospects of nanoscience with nanocrystals. ACS Nano.

[12] Wetterskog E (2016). Tuning the structure and habit of iron oxide mesocrystals. Nanoscale.

[13] Hanrath T (2012). Colloidal nanocrystal quantum dot assemblies as artificial solids. J. Vac. Sci. technol. A.

[14] Murray CB, Kagan CR, Bawendi MG (1995). Self-Organization of CdSe Nanocrystallites into Three-Dimensional Quantum Dot Superlattices. Science.

[15] Disch S (2013). Structural diversity in iron oxide nanoparticle assemblies as directed by particle morphology and orientation. Nanoscale.

[16] Biogini TP (2006). Kinetically driven self assembly of highly ordered nanoparticle monolayers. Phys. Rev. Lett..

[17] Narayanan S, Wang J, Lin X-M (2004). Dynamical Self-Assembly of Nanocrystal Superlattices during Colloidal Droplet Evaporation by *in situ* Small Angle X-Ray Scattering. Phys. Rev. Lett..

[18] Jiang Z, Lin X-M, Sprung M, Narayanan S, Wang J (2010). Capturing the Crystalline Phase of Two-Dimensional Nanocrystal Superlattices in Action. Nano Lett..

[19] Disch S (2011). Shape Induced Symmetry in Self-Assembled Mesocrystals of Iron Oxide Nanocubes. Nano Lett..

[20] Weidmann SC, Smiligies DM, Tisdale WA (2016). Kinetics of the self-assembly of nanocrystal superlattices measured by real-time *in situ* X-ray scattering. Nat. Mater..

[21] Bishop KJM, Wilmer CE, Soh S, Grzybowski BA (2009). Structural diversity in binary nanoparticle superlattices. Small.

[22] Siffalovic P (2007). Self-assembly of iron oxide nanoparticles studied by time-resolved grazing-incidence small-angle x-ray scattering. Phys. Rev. B.

[23] Rabani E, Reichman DR, Geissler PL, Brus LE (2003). Drying-mediated self-assembly of nanoparticles. Nature.

[24] Routh AF, Russel WB (1998). Horizontal drying fronts during solvent evaporation from latex films. AIChE J..

[25] Agthe M, Plivelic TS, Labrador A, Bergström L, Salazar-Alvarez G (2016). Following in Real Time the Two-Step Assembly of Nanoparticles into Mesocrystals in Levitating Drops. Nano Lett..

[26] Agthe M, Wetterskog E, Mouzon J, Salazar-Alvarez G, Bergström L (2014). Dynamic growth modes of ordered arrays and mesocrystals during drop-casting of iron oxide nanocubes. Cryst. Eng. Comm.

[27] Park J (2012). Direct Observation of Nanoparticle Superlattice Formation by Using Liquid Cell Transmission Electron Microscopy. ACS Nano.

[28] Woźniak M (2015). Formation of Highly Ordered Spherical Aggregates from Drying Microdroplets of Colloidal Suspension. Langmuir.

[29] Novotný F, Wandrol P, Pročka J, Šlouf M (2014). *In situ* WetSTEM observation of gold nanorod self-assembly dynamics in a drying colloidal droplet. Microsc. Microanal..

[30] Choi JJ, Bian K, Baumgardner WJ, Smilgies D-M, Hanrath T (2012). Interface-Induced Nucleation, Orientational Alignment and Symmetry Transformations in Nanocube Superlattices. Nano Lett..

[31] Roth SV (2007). *In situ* observation of nanoparticle ordering at the air-water-substrate boundary in colloidal solutions using x-ray nanobeams. Appl. Phys. Lett..

[32] Campolongo MJ (2011). Crystalline Gibbs Monolayers of DNA-Capped Nanoparticles at the Air-Liquid Interface. ACS Nano.

[33] Pietra F (2012). Semiconductor Nanorod Self-Assembly at the Liquid/Air Interface Studied by *in Situ* GISAXS and *ex Situ* TEM. Nano Lett..

[34] Vegso K (2011). *In situ* GISAXS monitoring of Langmuir nanoparticle multilayer degradation processes induced by UV photolysis. Phys. Stat. Sol. A.

[35] Siffalovic P (2008). Real-Time Tracking of Superparamagnetic Nanoparticle Self-Assembly. Small.

[36] Corricelli M (2014). GISAXS and GIWAXS study on self-assembling processes of nanoparticle based superlattices. Cryst. Eng. Comm.

[37] Geuchies JJ (2016). *In situ* study of the formation mechanism of two-dimensional superlattices from PbSe nanocrystals. Nat. Mater..

[38] Lu C, Akey AJ, Dahlman CJ, Zhang D, Herman IP (2012). Resolving the Growth of 3D Colloidal Nanoparticle Superlattices by Real-Time Small-Angle X-ray Scattering. J. Am. Chem. Soc..

[39] Radha B (2014). Reconstitutable Nanoparticle Superlattices. NanoLett..

[40] Routh AF (2013). Drying of thin colloidal films. Rep Prog Phys..

[41] Deegan RD (1997). Capillary flow as the cause of ring stains from dried liquid drops. Nature.

[42] Hu H, Larson RG (2006). Marangoni effect reverses coffee-ring depositions. J. Phys. Chem. B.

[43] Wetterskog E (2014). Precise control over shape and size of iron oxide nanocrystals suitable for assembly into ordered particle arrays. Sci. Technol. Adv. Mater..

[44] Faure B (2013). 2D to 3D crossover of the magnetic properties in ordered arrays of iron oxide nanocrystals. Nanoscale.

[45] Mueggenburg KE, Lin X-M, Goldsmith RH, Jaeger HM (2007). Elastic membranes of close-packed nanoparticle arrays. Nat. Mater..

[46] Josten, E. & Glavic, A. *In-situ* GISAXS software “FIT*in-situ*GISAXS”: https://github.com/science01/FITin-situGISAXS.

[47] Beverly KJ, Clint JH, Fletcher PDI (2000). Evaporation rates of structured and non-structured liquid mixtures. Phys. Chem. Chem. Phys..

