# Supplementary material for: Superlattice growth and rearrangement during evaporation-induced nanoparticle self-assembly
Source: Sci Rep. 2017 Jun 5;7:2802. doi: 10.1038/s41598-017-02121-4 (PMC5459806; doi:10.1038/s41598-017-02121-4)
Supplement: Supplementary file 1 — Supplementary Information [file 41598_2017_2121_MOESM1_ESM.pdf]

Superlattice growth and rearrangement during evaporation-induced nanoparticle self-assembly

Elisabeth Josten[1,2,*], Erik Wetterskog[4], Artur Glavic[1,5], Peter Boesecke[6], Artem Feoktystov[7], Elke Brauweiler-Reuters[8], Ulrich Rücker[1], German Salazar-Alvarez[3], Thomas Brückel[1], and Lennart Bergström[3]

[1]Jülich Centre for Neutron Science (JCNS) and Peter Grünberg Institute (PGI), JARA-FIT, Forschungszentrum Jülich GmbH, 52425 Jülich, Germany
[2]Institute of Ion Beam Physics and Materials Research, Helmholtz-Zentrum Dresden Rossendorf, 01328 Dresden, Germany
[3]Department of Materials and Environmental Chemistry, Stockholm University, 10691 Stockholm, Sweden
[4]Department of Engineering Sciences, Angström Laboratory, Uppsala University, 751 21 Uppsala, Sweden
[5]Laboratory for Neutron Scattering and Imaging, Paul Scherrer Institut, 5232 Villigen PSI, Switzerland
[6]ESRF-The European Synchrotron, 38043 Grenoble, France
[7]Jülich Centre for Neutron Science (JCNS) at Heinz Maier-Leibnitz Zentrum (MLZ), Forschungszentrum Jülich, 85747 Garching, Germany
[8]Institute for Complex Systems, Bioelectronics (ICS-8), Forschungszentrum Jülich GmbH, 52425 Jülich, Germany
[*]corresponding author:e.josten@hzdr.de

SI - Experimental

Small Angle X-ray Scattering SAXS and Grazing Incidence Small Angle X-ray Scattering GISAXS

Two different scattering geometries have been employed (Fig. SI-1); a transmission SAXS geometry with the substrate parallel to the incident beam and a standard GISAXS geometry with 0.3 ° angle of incidence. The incident beam with wave vector $\overrightarrow{k_i}$ ($|\overrightarrow{k}| = \frac{2\pi}{\lambda}$) is scattered at the sample and the scattered beam with wave vector $\overrightarrow{k_f}$ is detected. $\overrightarrow{Q} = \overrightarrow{k_f} - \overrightarrow{k_i}$ is the scattering vector and $\hbar\overrightarrow{Q}$ corresponds to the total momentum transferred during the scattering process. The area detector resolves the Q_y (perpendicular to the beam and parallel to the substrate, in-plane information) and Q_z (perpendicular to the substrate, out-of-plane information) directions.

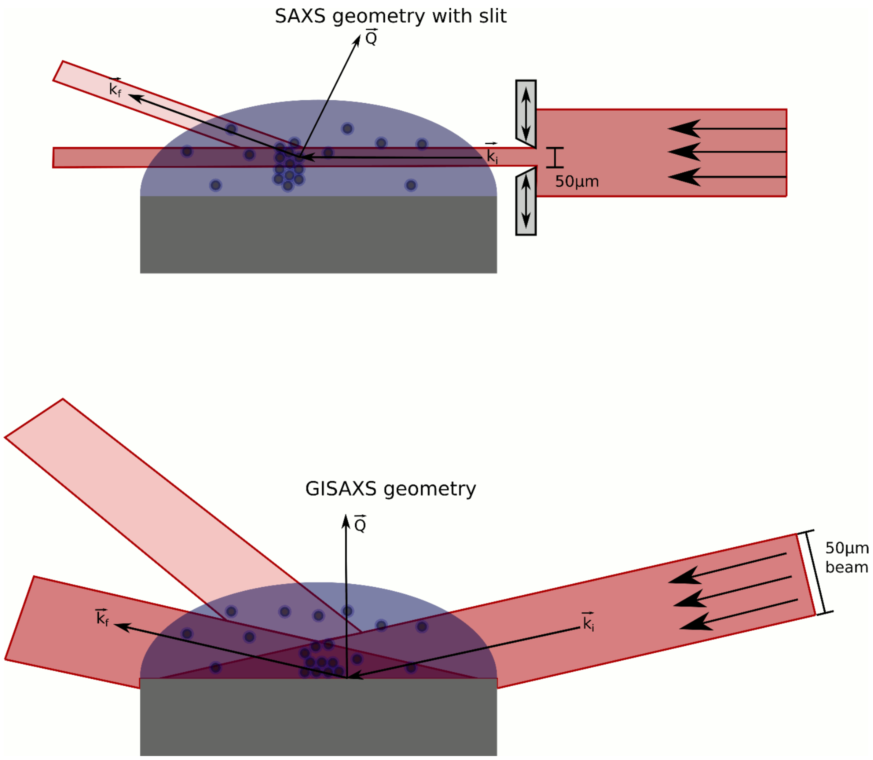

Figure SI - 1. Different geometries of the scattering experiment: A transmission SAXS geometry with the substrate parallel to the incident beam and a standard GISAXS geometry.

Data Evaluation

For a quantitative analysis of the scattering patterns (Fig. SI-2) the ensemble of 3D nanoparticle superlattices has been described in the framework of the Born approximation[1]. Dynamical scattering effects can be neglected for the given experimantal configuration except for Q_z position, which is corrected for dynamical scattering effects (see below). The positions of the peaks are given by the lattice parameters

$$Q_{HKL} = (H\frac{2\pi}{a}K\frac{2\pi}{b}L\frac{2\pi}{c}), \alpha = \beta = \gamma = 90°.$$

Peak intensities can be calculated from the single particle form factor and the selection rules for the $R\bar{3}m$ system. To accurately describe the peak shapes the instrumental resolution, nanoparticle superlattice size and nanoparticle superlattice ensemble variations of orientation and size need to be considered by a convolution of all three factors. In the actual implementation of our fit function the convolution is done using the Fast Fourier Transform (FFT) of the three shape functions. For the instrument resolution we use a window function with exponentially decaying wings (Equation 1) as empirical model to describe the beam

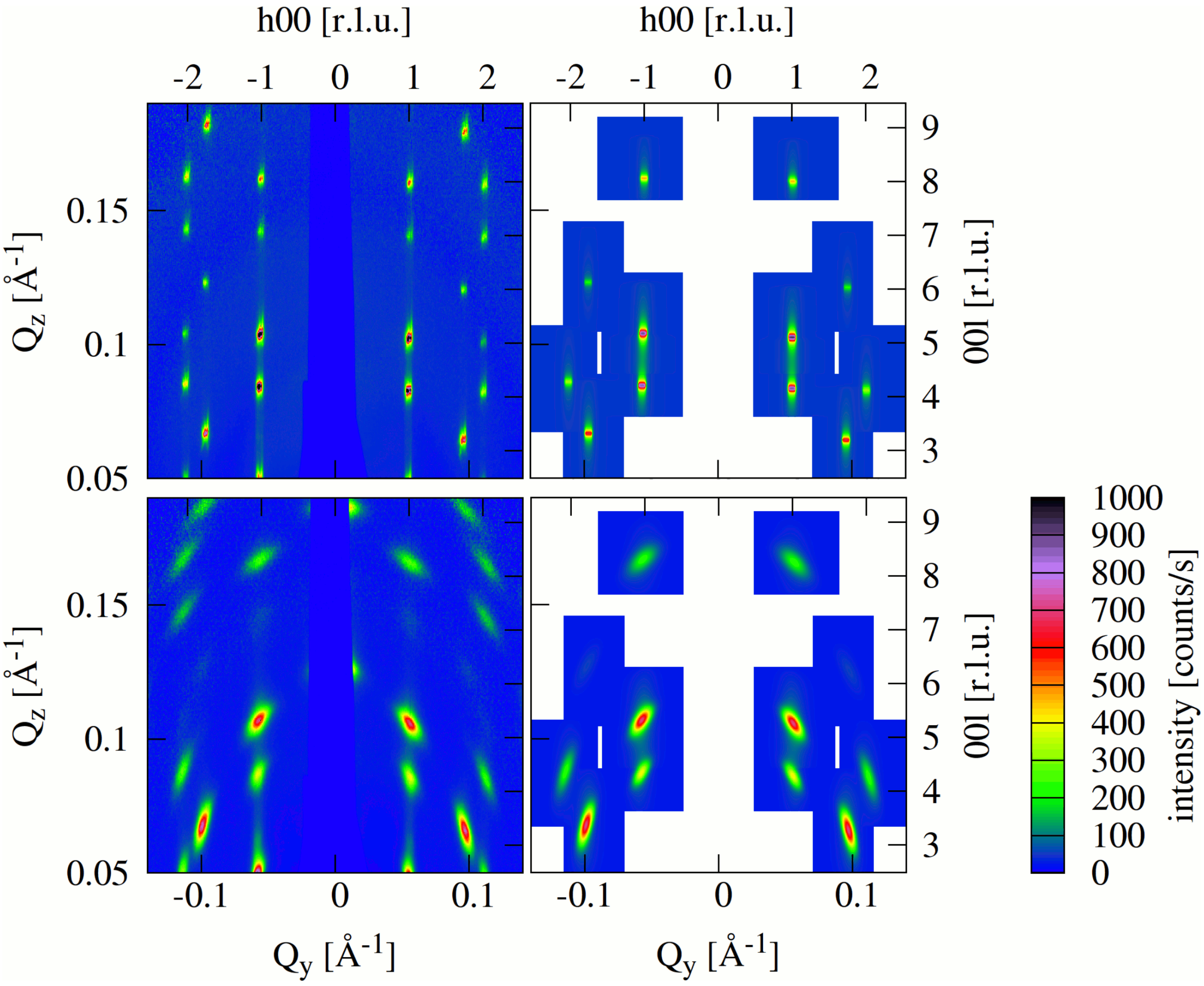

Figure SI - 2. Quality of the fit to the data. Measured (left) versus fitted (right) peak shapes as used for the time dependent data analysis.

width and pixel cross-talk.

$$B(x) = \begin{cases} e^{(x-\frac{w}{2})/d}, & \text{if } x < -\frac{w}{2} \\ e^{(\frac{w}{2}-x)/d}, & \text{if } x > \frac{w}{2} \\ 1, & \text{else} \end{cases} \tag{1}$$

The nanoparticle superlattice structural correlation lengths ξ_y, ξ_z are described by a 2D Lorentzian function, where the half width at half maximum is the inverse of the structural correlation in in-plane ($\gamma_y = \frac{2\pi}{\xi_y}$) and out-of-plane ($\gamma_z = \frac{2\pi}{\xi_z}$) directions.

$$L(\overrightarrow{Q}) = \frac{I_0}{1 + (\frac{\overrightarrow{Q_y}}{\gamma_y})^2 + (\frac{\overrightarrow{Q_z}}{\gamma_z})^2} \tag{2}$$

The random variation of the 3D nanoparticle superlattices' lattice parameters and the tilting of the 3D nanoparticle superlattices' c-axis against the substrate normal in an ensemble of 3D nanoparticle superlattices are modeled by a 2D Gaussian function in polar coordinates. The standard deviation in radial direction (σ_r) describes the variation of the lattice parameters

and the broadening in tangential direction (σ_t) describes the distribution of 3D nanoparticle superlattice tilt angles.

$$G(\overrightarrow{Q}) = e^{-\frac{1}{2}\frac{(\overrightarrow{Q_r}-r_o)^2}{r_o\cdot\sigma_r^2}} \cdot e^{-\frac{1}{2}\frac{(\phi-\phi_o)^2}{\sigma_t^2}}, \text{with} \quad \overrightarrow{Q_r} = \sqrt{\overrightarrow{Q_y}^2+\overrightarrow{Q_z}^2},\ \phi = \arctan(\frac{Q_z}{Q_y}) \tag{3}$$

Before fitting, the scattering patterns are treated as follows: every dataset is divided by the spherical particle form factor,[2] removing the common scaling factor for the Bragg peaks. Before scaling, a background from a detector region with no considerable scattering intensity is subtracted. The Q_z position of the peaks is corrected for refraction effects in the film according to the Distorted Wave Born Approximation (DWBA).[3] Other DWBA considerations can be neglected as the scattering patterns do not show any significant sign of Yoneda scattering or Bragg peaks from reflection at the substrate. These corrections allow a stable automatized fit routine over several hundred subsequent GISAXS images. The 6 most intense peaks ((018), (104), (015), (113), (024) and (116)) and their mirror reflections with negative Q_y value were chosen for the fitting process. This refinement procedure yields the lattice parameters, the in- and out-of-plane correlation lengths, and the size and tilt distribution widths. The refined parameters of the previous measurement are used as starting parameters for the subsequent pattern. The good agreement of the fit functions to the measured data is exemplified in Fig. SI-2.

SI - Results and discussion

SAXS and GISAXS

Fig. SI-3a represents an example SAXS measurement (*fast* evaporated sample). In all SAXS measurements no sign of agglomerates formation could be observed during the evaporation to a liquid film thickness of 230 micrometers. The SAXS data can be described well by a spherical form factor corresponding to well-dispersed nanoparticles with a radius of 50 $\mathring{A}$ at the beginning of the experiment (green line). The scattering signal does not show a considerable change during the evaporation until the total reflection from the droplet surface allows no further SAXS observation (red area). An estimate of the upper limit of the amount of particles that are involved in agglomerates formation is 5%. Such a mixture would produce a SAXS pattern equivalent to the sum of 95% particle form factor and 5% agglomerates structure factor (see Fig. 3b). For this estimate, a paste of the same particles, which forms short range closed packed structures, was measured at a lab source in SAXS geometry, normalized to the total scattering of the in-situ transmission measurement. After the droplet height has decreased to a level where the in-situ SAXS measurements could not be obtained any more, GISAXS datasets have been recorded continuously until all solvent has evaporated.

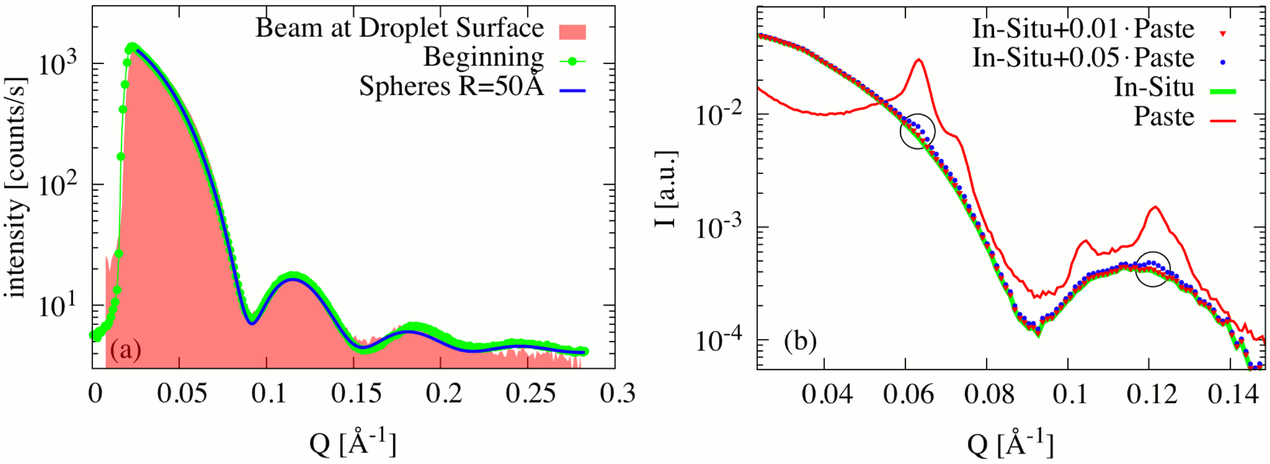

Figure SI - 3. X-ray scattering from the initial state. (a) SAXS measurements for a sample prepared with *fast* evaporation time showing no detectable change between the beginning of the experiment (green line) and the time where the height of the drop is at the beam height (red area). The blue line represents the theoretical form factor of a sphere with a radius of 50 $\mathring{A}$. (b) Estimate of the limit of detection of agglomerates. 5% of the particles present in agglomerates (blue circles) gives a visual clue as opposite to 1% (red triangles). The figure includes the pattern of a concentrated paste, which displays the presence of hard sphere agglomerates.

Camera view

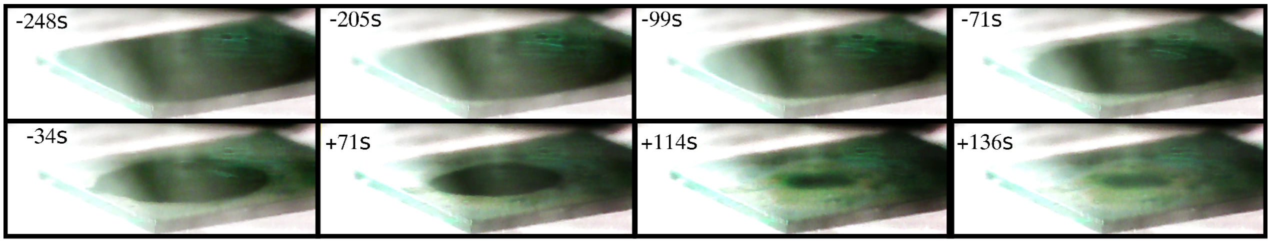

Figure SI - 4. Camera view. Time-dependent evolution of the movement of a drying front across the surface of the substrate (*slow* experiment).

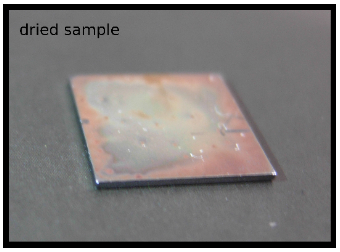

Figure SI - 5. Camera view of a dried sample. The sample is prepared with a *slow* evaporation rate. The scratches on the right side of the sample have been created by tweezers.

Lattice constant c/a ratio

In the rhombohedral structure ($R\overline{3}m$) a c/a ratio of 2.45 corresponds to a perfect *fcc* structure (indicated by the grey line in Fig. SI-6). The unit cell is extended in the *c* direction when nucleated, approaching the *fcc* packing during the superlattice shrinkage stage. At the end of the experiment, the *c*-axis is slightly contracted with respect to the *fcc* limit. In all cases the observation time was not long enough to reach a steady state, so the final values can only be estimated to be close to a perfect *fcc* structure.

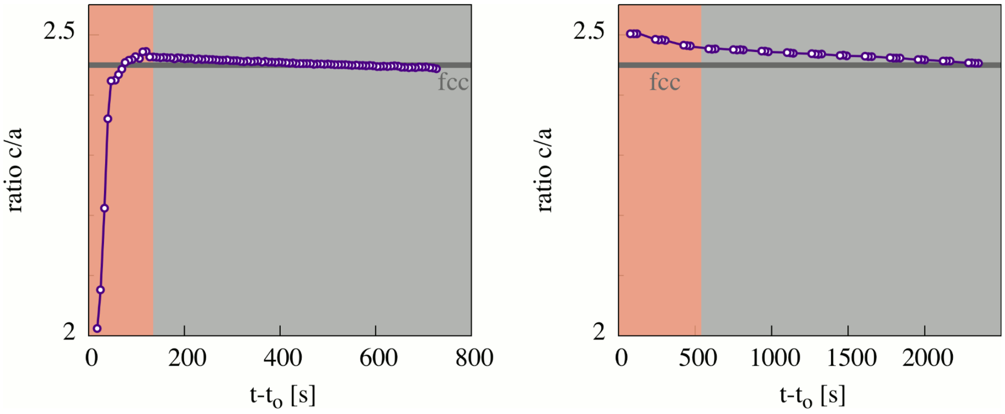

Figure SI - 6. Final structure close to *fcc* lattice. Lattice constant c/a ratio for the *fast* (left) and *slow* (right) evaporation rates.

References

1. Guinier, A. X-ray Diffraction. W.H. Freeman and Company ((1963)).
2. Pedersen, J. S. Analysis of small-angle scattering data from colloids and polymer solutions: modelling and least-squares fitting. *Adv. Coll. Inter. Sci* **70**, 171 (1997).
3. Busch, P. & Rauscher, M. & Smilgies, D.-M. & Posselt, D. & Papadakis, C. M. Grazing-incidence small-angle X-ray scattering from thin polymer films with lamellar structures - the scattering cross section in the distorted-wave Born approximation. *Appl. Cryst* **39**, 433 (2006).
